# Simulation-Driven Annotation-Free Deep Learning for Automated Detection and Segmentation of Airway Mucus Plugs on Non-Contrast CT Images

**DOI:** 10.3390/bioengineering13020153

**Published:** 2026-01-28

**Authors:** Lucy Pu, Naciye Sinem Gezer, Tong Yu, Zehavit Kirshenboim, Emrah Duman, Rajeev Dhupar, Xin Meng

**Affiliations:** 1 Department of Bioengineering, University of Pennsylvania, Philadelphia, PA 19104, USA; lpu@seas.upenn.edu; 2 Department of Radiology, Dokuz Eylül University, İzmir 35210, Türkiye; 3 Department of Radiology, University of Pittsburgh, Pittsburgh, PA 15213, USA; 4 Department of Bioengineering, University of Pittsburgh, Pittsburgh, PA 15213, USA; 5 Department of Cardiothoracic Surgery, Wake Forest University, Winston-Salem, NC 27109, USA

**Keywords:** mucus plug, non-contrast computed tomography, deep learning, simulation-driven, annotation-free, synthetic data generation, detection, segmentation

## Abstract

Mucus plugs are airway-obstructing accumulations of inspissated secretions frequently observed in obstructive lung diseases (OLDs), including chronic obstructive pulmonary disease (COPD), severe asthma, and cystic fibrosis. Their presence on chest CT is strongly associated with airflow limitation, reduced lung function, and increased mortality, making them emerging imaging biomarkers of disease burden and treatment response. However, manual delineation of mucus plugs is labor-intensive, subjective, and impractical for large cohorts, leading most prior studies to rely on coarse segment-level scoring systems that overlook lesion-level characteristics such as size, extent, and location. Automated plug-level quantification remains challenging due to substantial heterogeneity in plug morphology, overlap in attenuation with adjacent vessels and airway walls on non-contrast CT, and pronounced size imbalance in clinical datasets, which are typically dominated by small distal plugs. To address these challenges, we developed and validated a simulation-driven, annotation-free deep learning framework for automated detection and segmentation of airway mucus plugs on non-contrast chest CT. A total of 200 COPD CT scans were analyzed (98 plug-positive, 83 plug-negative, and 19 uncertain). Synthetic mucus plugs were generated within segmented airways by transferring voxel-intensity statistics from adjacent intrapulmonary vessels, preserving realistic morphology and texture while enabling controlled sampling of plug phenotypes. An nnU-Net trained exclusively on synthetic data (S-Model) was evaluated on an independent, expert-annotated test set and compared with an nnU-Net trained on manual annotations using 10-fold cross-validation (M-Model). The S-Model achieved significantly higher detection performance than the M-Model (sensitivity 0.837 [95% CI: 0.818–0.854] vs. 0.757 [95% CI: 0.737–0.776]; 1.91 false positives per scan vs. 3.68; *p* < 0.001), with performance gains most pronounced for medium-to-large plugs (≥6 mm). This simulation-driven approach enables accurate, scalable quantification of mucus plugs without voxel-wise manual annotation in thin-slice (<1.5 mm) non-contrast chest CT scans. While the framework could, in principle, be extended to other annotation-limited medical imaging tasks, its generalizability beyond this COPD cohort and imaging protocol has not yet been established, and future work is required to validate performance across diverse populations and scanning conditions.

## 1. Introduction

A mucus plug is an excessive buildup of mucus secretions within the airways, often found in patients with obstructive lung diseases (OLD), such as cystic fibrosis, asthma, and chronic obstructive pulmonary disease (COPD). Mucus plugging is strongly associated with cough, dyspnea, chest tightness, airflow obstruction, and reduced lung function [1,2,3]. Clinically, mucus plugging represents a key driver of disease burden. It exacerbates symptom severity, increases the frequency of acute exacerbations, and is linked to worse long-term outcomes, including elevated mortality [4,5,6,7,8]. For example, Diaz et al. [4,5,6,7,8] reported a nearly twofold increase in mortality among COPD patients with CT-visible mucus plugs. Consequently, mucus plugs are increasingly recognized both as targets for directed therapies [9,10] and as quantitative image biomarkers for assessing disease burden and treatment response [1,11,12]. Recent trials further highlight this therapeutic potential. For instance, treatment with Tezepelumab has been shown to reduce mucus plugging, improve lung function, and reduce inflammatory markers [13].

Despite these advances, automated tools for quantifying mucus plugs on chest CT remain limited. CT imaging is the standard modality for visualizing mucus plugs, yet manual identification is labor-intensive, subjective, and requires reviewing hundreds of slices across thousands of airway branches. As a result, most studies [1,2,13,14,15] rely on a segment-level mucus score as an index to characterize treatment efficacy and mucus plug burden. The score assigns one point per affected lung segment (e.g., one if one segment contains plugs regardless of the number, two if two segments, etc.). While practical, these coarse metrics do not capture lesion-level quantification [3]. In reality, mucus plugs vary widely in morphology, and their size and extent directly influence airflow obstruction. Fine-grained automated quantification tools are therefore needed to enable accurate burden assessment, longitudinal monitoring, and evaluation of emerging therapeutics.

Developing such automated methods remains challenging for two primary reasons. First, mucus plugs exhibit substantial heterogeneity in size, density, and distribution. Their CT attenuation often overlaps with that of adjacent vessels, airway walls, and small nodules, particularly on non-contrast scans where blood and mucus share similar radiomic densities (~30–50 HU). Second, and more fundamentally, clinical datasets are intrinsically imbalanced with respect to lesion size. In practice, small distal plugs are far more prevalent, whereas larger airway-obstructing plugs, despite their disproportionate impact on airflow limitation, occur relatively infrequently. As a result, deep learning models trained solely on real-world data tend to overfit to small, frequent findings and may fail to generalize to larger, more complex occlusions that are critical for disease severity and clinical outcomes. To date, only limited efforts have been made to develop automated algorithms for identifying mucus plugs on CT scans. Heng et al. [16] proposed a slice-level deep learning classification of mucus plugs in 2-D lung CT images, achieving a mean average accuracy of 0.55 in identifying which image slices have mucus plugs. However, this study did not attempt to segment mucus plugs automatically. Van der Veer et al. [17] used AI-based quantification but relied extensively on manual annotations and did not report model performance. Thus, a validated, automated, lesion-level mucus plug segmentation system is still lacking.

Deep learning offers strong potential for solving this problem, but requires large, diverse, and reliably annotated datasets. For mucus plug segmentation, annotated datasets are particularly scarce because plugs are infrequent, heterogeneous, and laborious to label. Synthetic data augmentation is a practical strategy for addressing data scarcity, and prior work shows that anatomically plausible synthetic examples can improve model robustness and generalization [18,19]. However, generic synthesis methods often overlook the anatomical and intensity constraints necessary for distinguishing mucus plugs from visually similar structures. Without task-specific anatomical fidelity, synthetic training data may fail to capture the subtle distinctions needed for accurate detection and segmentation.

To address the above challenges, we developed and validated a simulation-based deep learning framework for automated mucus plug detection and segmentation on non-contrast chest CT scans. Our approach leverages the fact that mucus plugs are always located within the airway and exhibit attenuation values similar to adjacent vessels due to their ~95% water composition [20]. We designed a targeted simulation pipeline that generates diverse, anatomically constrained mucus plugs inserted into real airway segments using intensity values sampled from neighboring pulmonary vessels. Crucially, this approach allows the training distribution to be controlled independently of the natural biological prevalence of mucus plugs. These synthetic examples increase the number and diversity of annotated positive instances in training while preserving realistic anatomical context. Importantly, synthetic data were used exclusively for model training, not for calibrating clinical prevalence. All evaluations were performed on independent test sets with manually annotated, real mucus plugs to ensure clinically valid performance assessment. This framework enables scalable, high-precision training without excessive burden on expert annotators and provides a path toward robust, clinically deployable mucus plug quantification.

## 2. Methods and Materials

### 2.1. Workflow Overview

**Figure 1:**
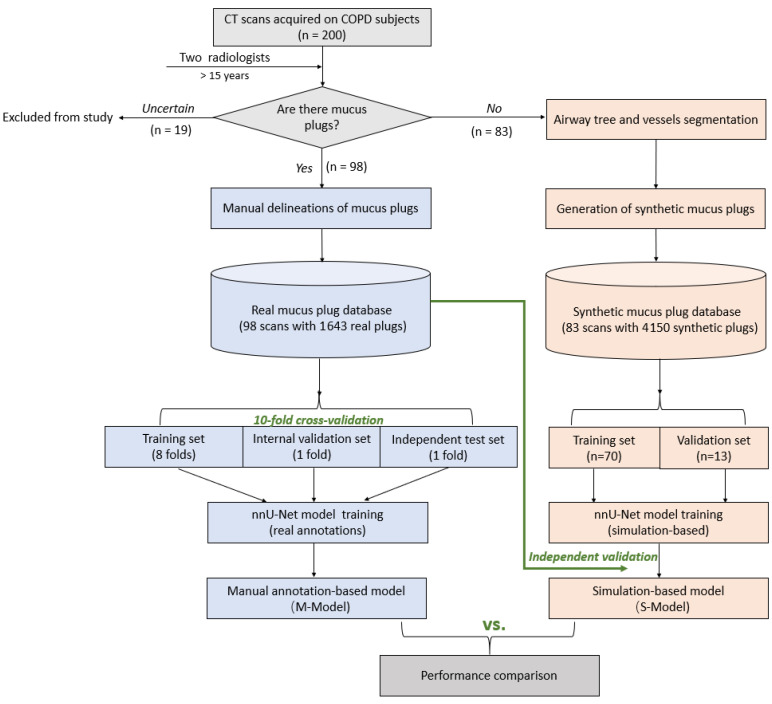
Workflow for the development and comparison of automated mucus plug detection and segmentation models. CT scans from 200 COPD subjects were reviewed by radiologists; 19 cases with uncertain findings were excluded from the study. The remaining scans were divided into two streams: (1) M-Model Development: Scans with confirmed mucus plugs (plug-positive, *n* = 98) were manually annotated to create a real mucus plug database, which was used to train the Manual annotation-based model (M-Model) using 10-fold cross-validation. (2) S-Model Development: Scans without mucus plugs (plug-negative, *n* = 83) were utilized to generate synthetic plugs. This synthetic database was used to train the simulation-based model (S-Model), which was subsequently validated against the real mucus plug database.

Figure 1 summarizes the end-to-end workflow of the proposed simulation-driven framework for automated mucus plug detection and segmentation. The pipeline consists of four major components: (1) CT scan categorization and expert annotation, (2) synthetic mucus plug generation, (3) automated detection and segmentation model training, and (4) quantitative model evaluation and comparison.

### 2.2. Chest CT Data and Expert Annotation

We analyzed 200 non-contrast chest CT scans from a COPD cohort [21]. All scans were de-identified and assigned study IDs prior to analysis. To ensure adequate visibility of small plugs, we included only series with slice thickness <1.5 mm; in-plane pixel size ranged from 0.549 to 0.738 mm. Two experienced thoracic radiologists independently reviewed all scans and, by consensus, classified them into three categories: mucus plug-positive (*n* = 98), mucus plug-negative (*n* = 83), and uncertain (*n* = 19), and annotated mucus plugs on these mucus plug-positive CT scans. Subject demographics are summarized in Table 1. Manual mucus plug segmentations were generated for all plug-positive scans using an in-house annotation tool [22]. These 98 expert-annotated scans served as the independent reference standard for evaluating the proposed model and as the dataset for training and cross-validation of manual-annotation-based benchmark model (M-Model). The 83 plug-negative scans served as the anatomical basis for generating synthetic mucus plugs, which constituted the reference standard for developing the proposed simulation-driven model (S-Model). The 19 uncertain scans were excluded from all stages of analysis, including training, validation, and testing. Subject demographics are summarized in Table 1.

### 2.3. Simulating Mucus Plugs in Chest CT Scans

**Figure 2:**
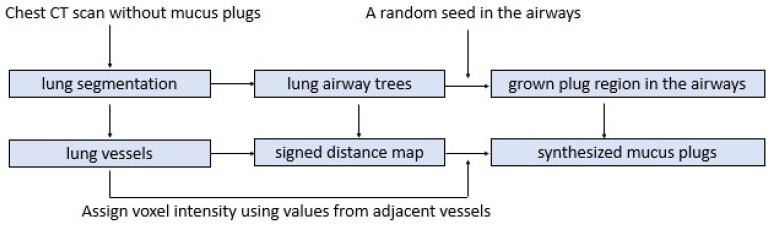
Schematic flowchart illustrating the pipeline used to simulate mucus plugs in the airways.

To generate training data without manual annotation, we developed a synthetic method that inserts simulated mucus plugs into the airway tree by replicating voxel intensities from neighboring pulmonary vessels. This design is based on two key observations: (1) mucus plugs occur exclusively within the airways, and (2) on non-contrast CT, their CT attenuation closely resembles that of adjacent vessels due to their high water content (~95%). A schematic overview of the algorithm is shown in Figure 2.

**Figure 3:**
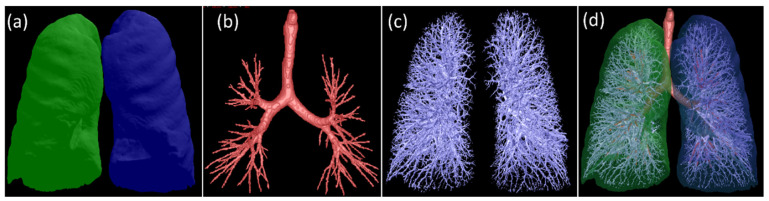
Example visualizations from a representative CT scan, including the segmented lung volumes (**a**), extracted airway tree (**b**), pulmonary vessels (**c**), and a combined rendering of all components (**d**).

Step 1: Anatomical segmentation. The lung volume, airway tree, and pulmonary vessels in the 83 mucus plug-negative CT scans were automatically segmented using in-house software [23,24,25,26] (Figure 3). Example segmentations are shown in Figure 3, including the segmented lung volumes (a), airway tree (b), pulmonary vessels (c), and a combined 3-D rendering (d).

Step 2: Region-growing to generate synthetic mucus plugs. A constrained region-growing algorithm was applied to simulate mucus plugs. A synthetic plug was initiated from a random seed point within the segmented airway lumen and iteratively expanded by adding neighboring voxels until the region satisfied a set of randomly sampled anatomical parameters: (a) plug length (2–20 mm): the longitudinal extent along the airway centerline; (b) occlusion ratio (0.35–1.0): the ratio of the plug’s cross-sectional area to the airway’s cross-sectional area, simulating conditions ranging from partial obstruction to complete blockage; and (c) airway size (1–20 mm): the diameter of the airways eligible for plug placement, ensuring plugs were distributed across both large bronchi and smaller distal airways. Random sampling across these parameters produced a heterogeneous set of synthetic mucus plugs with varying sizes, degrees of occlusion, and airway locations, thereby creating a heterogeneous set of synthetic plugs.

**Figure 4:**
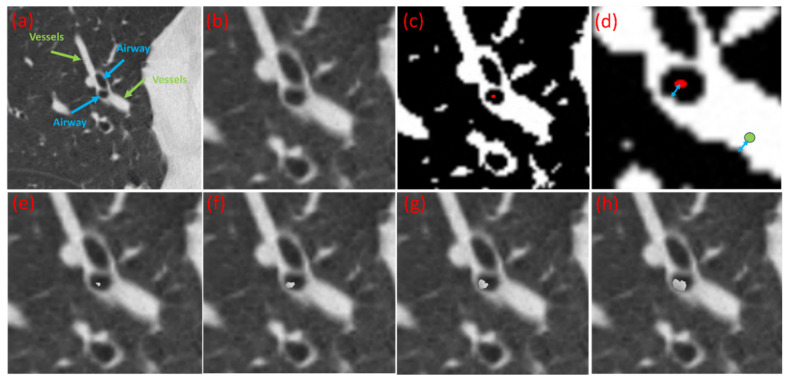
Illustration of the synthetic mucus plug generation algorithm. (**a**) Local region of a chest CT scan with airways (blue arrows) and pulmonary vessels (green arrows) is highlighted. (**b**) Magnified view of the same region. (**c**) Initialization of a random seed point (red dot) within the segmented airway lumen. (**d**) Spatial mapping step showing the corresponding point selected in a neighboring vessel (green circle) such that its signed distance to the vessel wall matches the signed distance of the airway seed to the airway wall (equality indicated by blue arrows). (**e**–**h**) Progressive stages of the region-growing process. The synthetic plug (grey) begins at the airway seed point and expands iteratively, inheriting voxel intensities from the matched vessel location while conforming to the airway’s anatomical constraints.

Step 3: Spatial mapping via signed distance fields. To ensure realistic intensity texture, we established a spatial correspondence between the airway lumen and adjacent vessels. For any given voxel *P_airway_* within the generated plug region (red dot in Figure 4c), we computed the signed distance field (SDF), representing the minimum distance from *P_airway_* to the airway boundary (Figure 4d, blue arrow beginning at the red dot). The SDF was computed using the fast marching method (FMM) [27] due to its O(*N* log *N*) computational efficiency and high accuracy in capturing distances within thin tubular structures. The sign of the distance indicates whether the point lies inside (negative) or outside (positive) the boundary, providing a precise quantitative measure of anatomical depth.

**Figure 5:**
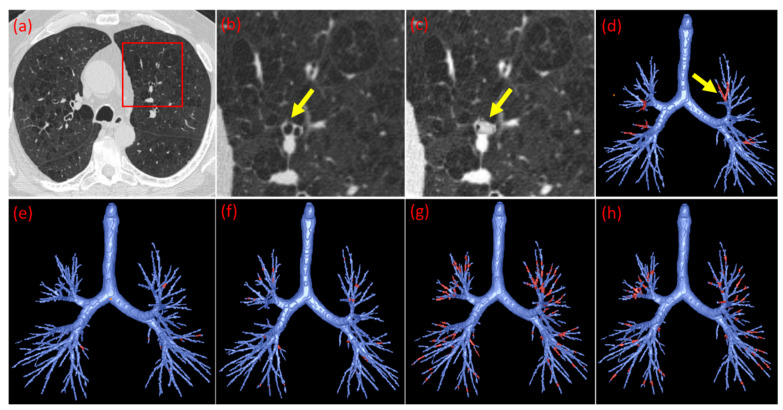
Examples of synthetic mucus plugs generated for training. (**a**) Axial slice of a plug-negative CT scan. (**b**) Magnified view of the targeted airway highlighted by the yellow arrow prior to modification. (**c**) The same airway after insertion of a synthetic mucus plug. The plug is strictly confined to the lumen, with voxel intensities mapped from adjacent vessels to ensure realistic texture. (**d**) Three-dimensional rendering of airway tree (blue) with synthetic mucus plugs visualized in red (yellow arrow indicates the plug corresponding to panels (**b**,**c**)). (**e**–**h**) Three-dimensional illustration of the stochastic diversity of the training set. These examples demonstrate how the algorithm varies the number, location, and dimensions of plugs across different samples to ensure robust machine learning training.

Step 4: Intensity transfer. For each voxel in the synthetic plug, an adjacent vessel of comparable caliber was identified. A corresponding vessel voxel *P_vessel_* vessel was selected such that its signed distance to the vessel wall matched the signed distance of the airway voxel *P*_airway_ to the airway wall (Figure 4d). This geometric isomorphism ensures that the synthetic plug inherits a realistic density profile (Hounsfield Units) consistent with the partial volume effects and noise texture of the surrounding tissue specific to non-contrast imaging. The intensity value of *P_vessel_* was then mapped to *P_airway_*. This process was repeated iteratively for all voxels in the region (Figure 4e–h), resulting in a dataset of synthesized plug-positive scans (Figure 5).

Step 5: Training data construction. This synthesis pipeline enables the precise definition of synthetic “ground truth” labels without manual annotation. To train the Convolutional Neural Network (CNN), we constructed mini-batches containing a fixed ratio of synthetic plug-positive patches and original plug-negative patches. This balance prevents the model from overfitting to synthetic artifacts and ensures robust performance on real-world data. The optimization of this positive-to-negative ratio is detailed in Section 2.5 (Performance evaluation).

### 2.4. CNN-Based Mucus Plug Detection and Segmentation

Prior to model training, all CT images and their corresponding annotation masks were resampled to an isotropic resolution of 0.8 × 0.8 × 0.8 mm^3^ to ensure spatial consistency. We employed the nnU-Net framework [28] for automated detection and segmentation. nnU-Net is a self-adapting implementation of the U-Net architecture [29] selected for three reasons: (1) It automatically determines optimal pipeline hyperparameters, including network topology, patch size, and preprocessing strategies based on the dataset properties. (2) The framework has demonstrated superior segmentation accuracy compared to specialized architectures across 23 public datasets [16,28]. (3) It provides a standardized, reproducible architecture, allowing the study to focus on testing and validating our simulation-based procedures rather than developing novel CNN architectures. To validate the simulation pipeline, we trained two distinct models: the S-Model, trained on mucus plug-negative CT scans augmented with synthetic plugs, and the M-Model, trained on mucus plug-positive CT scans with manually annotated real mucus plugs.

### 2.5. Performance Evaluation

We first conducted experiments to determine the optimal number of synthetic plugs to insert per CT scan. This step was crucial to ensure the model received sufficient synthetic “ground truth” examples (airway regions with synthetic plugs) without overwhelming the “negative” background (airway regions without plugs). To calibrate this balance, we assessed S-Model performance by varying the number of synthetic plugs per scan from 10 to 100, in increments of 10. The locations and dimensions of these plugs were randomized to ensure diversity. This optimization was performed solely to calibrate the training data generation; all subsequent evaluations were conducted on real clinical data.

Model performance was evaluated as follows: (1) S-Model: Trained on the 83 plug-negative CT scans, each augmented with the optimal number of synthetic plugs determined in the previous step. This model was tested on an independent dataset of 98 plug-positive CT scans. (2) M-Model: Trained and validated on the 98 plug-positive CT scans using 10-fold cross-validation. Hereafter, the 98 plug-positive CT scans are referred to as the independent test set for evaluating the S-Model and as the cross-validation dataset for training and evaluating the M-Model.

Performance was quantified using both pixel-level and object-level metrics. The Dice Similarity Coefficient [30] measured the volumetric overlap between the predicted segmentation (A) and the ground truth annotation mask (B) at the pixel level:

(1) $$D(A,B)=\frac{2\left|A\cap B\right|}{\left|A\right|+\left|B\right|}$$

Sensitivity (true positive rate) and False Positives per CT scan were computed to assess detection capability at the mucus plug region level. A predicted mucus plug region was considered a true positive (TP) if it overlapped with the radiologist-annotated mucus plug by at least one voxel. A false positive (FP) was defined as a predicted mucus plug region that did not overlap with any annotated region. A false negative (FN) corresponded to the radiologist-annotated mucus plug for which no overlapping predicted region was identified.

Consistent with prior methodologies [21,29], Receiver Operating Characteristic (ROC) analysis was not utilized. Since the employed nnU-Net inference pipeline yields binary segmentation masks without explicit object-level confidence scores, varying detection thresholds to compute ROC curves and AUC metrics was not feasible.

Statistical comparisons of performance metrics between models were conducted using paired, two-sided Student’s *t*-tests. Statistical significance was defined as *p*-value < 0.05.

## 3. Results

### 3.1. Optimization of Synthetic Plug Augmentation Count

**Figure 6:**
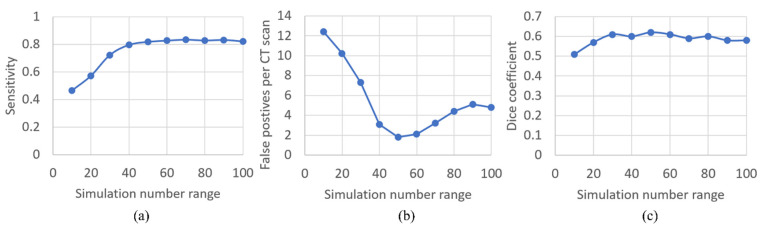
Impact of synthetic mucus plug count on S-Model performance. Graphs display (**a**) sensitivity, (**b**) false positives per CT scan, and (**c**) Dice similarity coefficient as a function of the synthetic plug count inserted per CT scan. Performance metrics stabilized at an optimal density of 50 plugs per scan.

We analyzed how the density of synthetic augmentation (number of plugs inserted per scan) influenced S-Model performance. As shown in Figure 6a, the sensitivity of nnU-net increased rapidly as the augmentation density increased from 10 to 40 plugs per scan, then stabilized at approximately 0.82 (95% CI: 0.79–0.85) when the count reached 50. Conversely, the false positive rate exhibited a U-shape trajectory (Figure 6b). It decreased substantially as the number of synthetic plugs increased, reaching a minimum at 50 plugs per scan. Beyond this point, false positives began to rise, likely due to an imbalance where the model became over-sensitized to plug-like features relative to the airway background. The Dice coefficient (Figure 6c) showed minimal variation once the synthetic plug count exceeded 30 per scan, suggesting that segmentation accuracy was relatively insensitive to further increases. These trends indicate that inserting ~50 synthetic plugs per scan provides sufficient diversity to supply plug-positive patches for training while avoiding degradation in false positive performance. Accordingly, 50 synthetic plugs per CT scan were selected as the optimal augmentation count. Applying this to the 83 mucus plug-negative CT scans generated a total of 4150 synthetic plugs, with their characteristics summarized in Table 2.

### 3.2. Summary of the Synthetic and Real Mucus Plugs in the Study Cohort

Table 2 summarizes the morphological characteristics of the manually annotated (real) and algorithmically generated (synthetic) mucus plugs. The real mucus plugs (*n* = 1643) were manually delineated from 98 mucus plug-positive CT scans, whereas the synthetic mucus plugs were generated from 83 plug-negative CT scans using the proposed simulation pipeline. The real mucus plugs exhibit significant heterogeneity in count, volume, and length. The number of plugs per scan ranged from 1 to 146, reflecting highly variable disease burden across patients. Most of these real plugs were relatively small, with 46.9% having a length of less than 3 mm. Only 17.0% fell within the 6–15 mm range. In contrast, the number of synthetic mucus plugs (*n* = 4150) was 2.5 times greater than the number of real mucus plugs. These synthetic mucus plugs exhibit less heterogeneity in density, volume, radius, and length. However, there are variations in the length distributions; specifically, 63.9% of the synthetic mucus plugs fall within the length range of [3, 6), whereas 46.9% of real mucus plugs measure less than 3 mm in length. Importantly, although the relative proportion of large plugs (>15 mm) was similar between the real and synthetic datasets (approximately 8%), the simulation pipeline generated substantially more large plugs in absolute terms, with 328 synthetic instances compared with 134 manually annotated plugs, representing a 2.4-fold increase in the number of unique large plug examples.

A critical advantage of the synthetic pipeline is time efficiency. Manual delineation by an experienced radiologist required 20 to 250 min per CT scan, depending on the number of mucus plugs present. In contrast, the fully automated simulation pipeline, including lung vessel and airway segmentation followed by plug generation, required only about five minutes per scan. This represents a dramatic reduction in workload while offering precise control over training data parameters such as count, density, and occlusion ratio.

### 3.3. Performance Comparison: S-Model vs. M-Model

The performance metrics for the simulation-based model (S-Model) and the manual-annotation-based model (M-Model) are summarized in Table 3. Overall, the S-Model demonstrated superior performance for both detection and segmentation. On the independent test set of 98 mucus plug-positive CT scans (containing real mucus plugs), the S-Model achieved a sensitivity of 0.837 (95% CI: 0.81800.854), significantly higher than the M-Model’s 0.757 (95% CI: 0.737–0.776) (*p*-value < 0.001). The S-Model also reduced the false-positive rate by nearly 50%, averaging 1.91 false positives per scan compared with 3.68 for the M-Model. Regarding segmentation accuracy, the S-Model achieved a higher overall Dice coefficient (0.631) compared to the M-Model (0.557), indicating better volumetric overlap with the radiologist-defined ground truth.

Stratified analysis by plug size revealed that the performance gap widened as mucus plug size increased. For small Plugs (<3 mm), the S-Model achieved 0.789 (95% CI: 0.762–0.814), comparable to the M-Model at 0.794 (95% CI: 0.768–0.819). For medium plugs [3–6 mm), the S-Model sensitivity was 0.843 (95% CI: 0.808–0.873) versus 0.725 (95% CI: 0.686–0.761) for the M-Model. For medium–large plugs [6–15 mm), the S-Model achieved 0.907 (95% CI: 0.868–0.938), outperforming the M-Model at 0.736 (95% CI: 0.678–0.788). For the largest plugs (>15 mm), the S-Model achieved 0.940 (95% CI: 0.884–0.973), while the M-Model reached only 0.694 (95% CI: 0.606–0.771).

**Figure 7:**
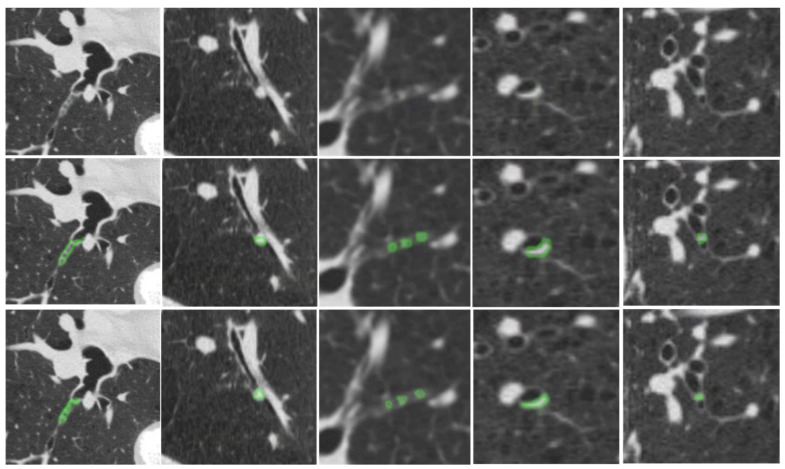
Examples illustrating the detection and segmentation performance of the S-Model compared with manual annotation. Top row: original CT images. Middle row: manually annotated ground truth mucus plugs (green). Bottom row: S-Model predictions (green). Notably, the M-Model failed to detect these mucus plugs.

**Figure 8:**
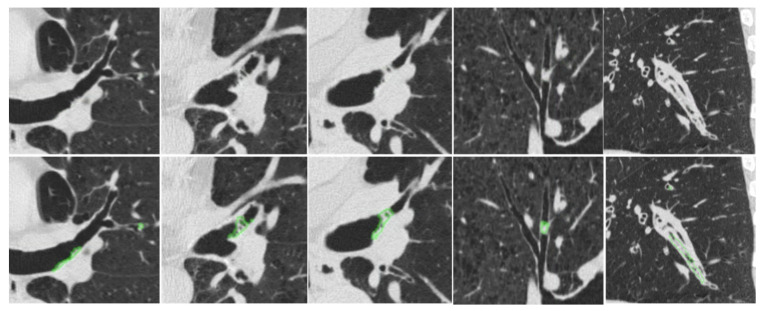
Examples of mucus plugs detected and segmented by the S-Model but missed by the M-Model. Top row: original CT images. Bottom row: S-Model predictions (green).

**Figure 9:**
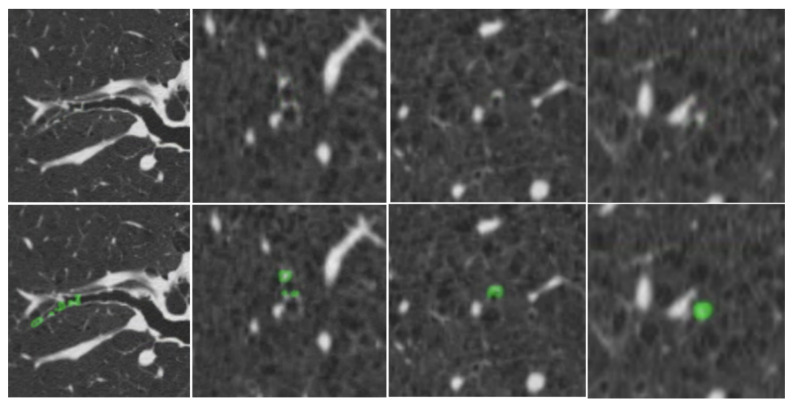
Examples of false negatives where both the S-Model and M-Model failed to detect the manually annotated regions. These misses typically occur in low-contrast regions or very small distal airways. Top row: original CT images. Bottom row: manually annotated mucus plugs (green).

**Figure 10:**
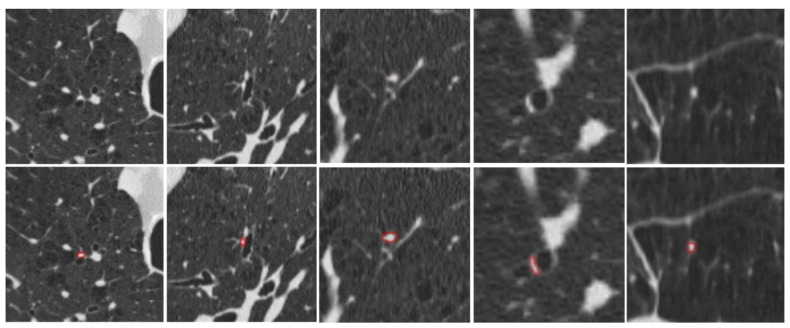
Examples of false positive detections generated by the S-Model. Top row: original CT images. Bottom row: S-Model prediction (red). These errors predominantly stem from small adjacent vessels or airway wall thickening that mimics the density profile of mucus.

Figure 7 provides qualitative examples of the S-Model’s high segmentation fidelity. Figure 8 illustrates cases where the S-Model successfully detected large plugs that were missed by the M-Model. However, shared limitations remain; Figure 9 displays false negatives common to both models, typically occurring in distal airways with low contrast. Figure 10 illustrates typical S-Model false positives, which primarily arose from small pulmonary vessels or remodeled airway walls that mimic plug-like attenuation and morphology.

Both nnU-Net models were able to automatically detect and segment mucus plugs on a single CT scan within 2–3 min on a standard NVIDIA RTX 3090 GPU.

## 4. Discussion

Motivated by the emerging role of mucus plugging as a therapeutic target in obstructive lung diseases (OLD) [1,2,3,4,5,6,7,8], we developed and validated a novel simulation-based deep learning framework for automated detection and segmentation. The central innovation of this study lies in decoupling data generation from model training. Through a simulation-based training framework, we eliminate the bottleneck of labor-intensive manual annotation while generating high-fidelity, anatomically realistic training data that capture the heterogeneous manifestations of mucus plugging.

Mucus plugs exhibit substantial phenotypic variability in size, shape, and spatial distribution. Models trained solely on real clinical data tend to be biased toward common presentations, often underrepresenting rare or extreme patterns of airway occlusion. The simulation-based approach developed here overcomes this limitation by stochastic parameterization and systematic variation in plug length, occlusion ratio, and airway caliber. This design ensures that the model is exposed to a wide spectrum of anatomically plausible scenarios during training. Our experiments demonstrated that the S-Model (trained on synthetic data) consistently outperformed the M-Model (trained on real data), particularly for clinically significant plugs larger than 3 mm. These findings suggest that the synthetic dataset provided a broader and more representative feature space than the limited manually annotated dataset. Moreover, the typical “simulation-to-reality” gap, which is often a major challenge in synthetic training, was minimized by two factors: (1) the inherent radiomic similarity between mucus and blood, both of which exhibit high water content and similar attenuation characteristics; and (2) the application of dynamic data augmentation (e.g., Gaussian smoothing, noise injection) to emulate realistic image acquisition variability.

This performance difference can be partly explained by a size-related imbalance in the training data. Models trained solely on real clinical data tend to be biased toward the most common presentation—small, distal plugs (<3 mm), which constituted 46.9% of our manually annotated dataset. This over-representation of tiny, dot-like features may limit the model’s ability to learn discriminative representations for large, tubular obstructions. In contrast, the synthetic dataset shifted the training distribution toward more distinct tubular structures, with 63.9% of synthetic plugs falling in the 3–6 mm range. Moreover, although the relative proportion of large plugs (>15 mm) was similar across datasets, the synthetic pipeline generated 2.4 times more unique examples of these plugs (328 synthetic vs. 134 real). This enrichment in absolute training volume likely enabled the S-Model to learn more robust features for large, complex airway casts that were underrepresented in manual annotations, plausibly explaining the progressive performance gains observed for medium-to-large plugs (≥6 mm), including the largest airway-occluding casts.

One performance divergence was observed in small mucus plugs (<3 mm), where the M-Model showed slightly higher sensitivity than the S-Model (Table 3). This attribution is likely structural rather than methodological. The simulation-based algorithm relies on a prerequisite airway segmentation map to “seed” the plugs. If the underlying segmentation algorithm fails to resolve distal sub-segmental airways (a known challenge in CT imaging), the synthesis pipeline cannot generate training examples in those locations. Consequently, the S-Model may be under-exposed to plugs in the smallest airway generations compared to the M-Model, which learned from human annotators who can visually identify plugs even where automated airway segmentation fails. Future iterations could mitigate this limitation by incorporating additional plug-negative scans with higher-resolution airway trees or by oversampling the generation of small distal plugs to enrich the training distribution.

The Dice coefficients for small plugs (<3 mm) were relatively low (e.g., 0.475). It is well-established in medical image computing that Dice scores are sensitive to object size; for small structures, a single-voxel segmentation error causes a disproportionate drop in the metric due to the high surface-to-volume ratio. Therefore, Dice alone is an insufficient metric for clinical utility. When evaluated by detection metrics (Sensitivity and False Positives per scan), the S-Model demonstrated robust performance (Sensitivity: 0.837, FP: 1.91), proving its ability to accurately localize pathology even if the voxel-wise boundary definition varies slightly. Consistent with prior segmentation studies [22,31], ROC analysis was not utilized. Because the nnU-Net pipeline outputs binary segmentation masks rather than object-level probability scores, varying detection thresholds to generate an ROC curve is not methodologically feasible. The sensitivity and false positives per scan provide the direct and appropriate assessment of the system’s clinical detection capability.

While we employed nnU-Net due to its self-configuring architecture, the core contribution of this work is the simulation methodology itself. This modular framework creates annotation-free training data by combining anatomical constraints with voxel intensity mapping from adjacent vessels. This approach is agnostic to the downstream network architecture; it can be integrated with any current or future CNN, allowing researchers to augment existing models with diverse, realistic examples. This flexibility positions the simulation strategy as a versatile tool for scaling up automated analysis in pulmonary imaging without the constraints of manual data curation.

We acknowledge several limitations of this study. First, the algorithm requires chest CT scans that are confirmed to be free of mucus plugs. While this greatly reduces the need for labor-intensive manual annotation, it still requires expert review to ensure that the selected scans are truly negative. Second, the method is currently limited to thin-slice CT scans (<1.5 mm), as mucus plugs are not reliably visualized at thicker reconstructions. Consequently, model performance may not generalize to thicker-slice scans. However, thin-slice acquisitions are standard in modern clinical chest CT protocols, especially in studies related to mucus plugs, so this limitation is unlikely to pose a significant barrier to practical adoption. Third, the dataset used for model development and validation, particularly the independent real-world validation set, was relatively small. This reflects the inherent challenge of assembling large, well-annotated datasets for mucus plugs, which are highly heterogeneous and often small in size. Nonetheless, the strong performance achieved despite this limitation highlights the promising potential of the proposed method. Fourth, inter-reader agreement was not computed, as voxel-level annotations were refined through repeated consensus to establish the reference standard for training and validation. Future work could benefit from explicitly assessing inter-observer variability. Finally, although the proposed simulation-driven framework is conceptually extensible, the current study validates performance only for mucus plug detection and segmentation on thin-slice, non-contrast chest CT scans in a COPD cohort, and generalizability to other diseases, imaging protocols, or institutions has not yet been established.

## 5. Conclusions

We developed and validated a simulation-driven deep learning framework for automated detection and segmentation of airway mucus plugs on thin-slice, non-contrast chest CT scans. By synthesizing anatomically constrained mucus plugs directly within the airway tree, the proposed approach eliminates the need for voxel-level manual annotation while generating diverse, realistic training data that capture key phenotypic variations in mucus plugging. When evaluated on an independent, expert-annotated COPD test set, the simulation-trained model demonstrated robust detection and segmentation performance and outperformed a model trained exclusively on real annotations, particularly for clinically relevant medium-to-large plugs. Notably, the current validation is confined to COPD cohorts and standardized thin-slice CT acquisitions; generalizability to other obstructive lung diseases, imaging protocols, or multi-center settings has not yet been established. Future work will focus on external, multi-institutional validation, evaluation in asthma and cystic fibrosis populations, assessment across heterogeneous acquisition parameters, and longitudinal studies to determine clinical utility for disease phenotyping and treatment monitoring.

## Figures and Tables

**Table 1:** Subject demographics and disease characteristics in this study (*n* = 200).

	All Subjects (*n* = 200)	Male (*n* = 120)	Female (*n* = 80)
Age, Mean (Range)	66.9 (46–82)	67.4 (50–82)	66.1 (46–79)
Race, *n* (%)			
White	189 (94.5)	114 (95.0)	75 (93.8)
African American	10 (5.0)	5 (4.2)	5 (6.2)
Other races	1 (0.5)	1 (0.8)	0 (0)
Weight, kg, mean (SD)	81.4 (16.4)	88.1 (15.5)	71.8 (12.3)
Height, cm, mean (SD)	170.0 (9.5)	175.7 (6.7)	161.9 (6.4)
COPD Severity			
Normal	76	42	34
Mild	35	20	15
Moderate	59	38	21
Severe	20	14	6
Very severe	10	6	4
Mucus plug status, *n* (%)			
Yes	98 (49.0)	62 (51.7)	36 (45.0)
No	83 (41.5)	48 (40.0)	35 (43.8)
Uncertain	19 (9.5)	10 (8.3)	9 (11.2)

**Table 2:** Summary of the characteristics of the real and synthetic mucus plugs.

	Real Mucus (in 98 CT Scans)	Synthetic Mucus (in 83 CT Scans)
Count: total	1643	4150
mean (SD), range, per CT scan	17.1 (26.5), 1–146	50 (0), 50
Volume (mm^3^): mean (SD), range	49.35 (220.0), 1.47–2136.28	46.86 (45.8), 1.35–530.2
Length (mm): mean (SD), range	6.06 (6.89), 1.14–29.75	8.90 (4.62), 1.35–27.76
Distribution based on length:		
(0, 3)	770 (46.9%)	768 (18.5%)
[3, 6)	459 (27.9%)	2652 (63.9%)
[6, 15)	280 (17.0%)	402 (9.7%)
[15, ∞)	134 (8.2%)	328 (7.9%)

SD—standard deviation.

**Table 3:** Performance comparison between the S-Model and M-Model evaluated on the same set of 98 mucus plug-positive CT scans. Values represent Mean (Standard Deviation) or Rate (Count).

Mucus Size (Length: mm)	Metrics	S-Model	M-Model
(0, 3)	Dice coefficient	0.475 ± 0.145	0.482 ± 0.132
	Sensitivity	0.789 (608/770; 95% CI: 0.762–0.814)	0.794 (611/770; 95% CI: 0.768–0.819)
	False positives per scan	1.34 (131/98)	1.21 (119/98)
[3, 6)	Dice coefficient	0.520 ± 0.099	0.494 ± 0.113
	Sensitivity	0.843 (387/459; 95% CI: 0.808–0.873)	0.725 (333/459; 95% CI: 0.686–0.761)
	False positives per scan	0.378 (37/98)	1.04 (102/98)
[6, 15)	Dice coefficient	0.672 ± 0.082	0.610 ± 0.109
	Sensitivity	0.907 (254/280; 95% CI: 0.868–0.938)	0.736 (206/280; 95% CI: 0.678–0.788)
	False positives per scan	0.204 (20/98)	0.643 (63/98)
[15, ∞)	Dice coefficient	0.777 ± 0.080	0.694 ± 0.118
	Sensitivity	0.940 (126/134; 95% CI: 0.884–0.973)	0.694 (93/134; 95% CI: 0.606–0.771)
	False positives per scan	0 (0/98)	0.786 (77/98)
All	Dice coefficient	0.631 ± 0.088	0.557 ± 0.119
	Sensitivity	0.837 (1375/1643; 95% CI: 0.818–0.854)	0.757 (1243/1643; 95% CI: 0.737–0.776)
	False positives per scan	1.91 (188/98)	3.68 (361/98)

## Data Availability

The original contributions presented in the study are included in the article, further inquiries can be directed to the corresponding author.
